# Starts and Stops: Strategizing an Age-Friendly University Commitment During a Pandemic

**DOI:** 10.1093/geroni/igab046.2802

**Published:** 2021-12-17

**Authors:** Paige Ebner, Kenneth Ferraro, Brian Pastor, Wendy Rogers

**Affiliations:** 1 Purdue University, West Lafayette, Indiana, United States; 2 University of Illinois Urbana-Champaign, Champaign, Illinois, United States

## Abstract

The Global Network of Age-Friendly Universities seeks to enhance age-inclusivity and engagement in higher education, but delivering age-friendly programming became very challenging during the COVID-19 pandemic. We examine how two land-grant universities adapted to the pandemic and draw some lessons from those experiences that may be useful for other universities seeking to implement or resume the AFU programming. The two main responses were to either pause many of the age-friendly initiatives at the university or adapt to virtual or online delivery platforms. To ensure the health and safety of older adults, colleges and universities paused many age-friendly initiatives such as intergenerational service-learning, technological assistance to older adults, and influenza vaccinations. Other programs continued but in a modified delivery format. Examples include: converting a face-to-face balance-training program to telehealth delivery; transitioning visitation programs to pen pal communication; and replacing face-to-face workshops offered by Extension Services with webinar delivery. Despite these challenges, we conclude that moving to virtual platforms and other methods of delivery, including conventional mail, has in some cases increased access for many older adults and became a lifeline during a time of social isolation for many older adults. Taken together, these experiences highlight the need for age-friendly universities to have contingency plans to ensure continuation of age-friendly programming in the event of pandemics or disasters. Finally, the pause in programming creates opportunities to re-launch or re-organize those initiatives in accord with federal and state safety guidelines.

